# Applying near-infrared photoimmunotherapy to B-cell lymphoma: comparative evaluation with radioimmunotherapy in tumor xenografts

**DOI:** 10.1007/s12149-017-1197-9

**Published:** 2017-07-24

**Authors:** Yusri-Dwi Heryanto, Hirofumi Hanaoka, Takahito Nakajima, Aiko Yamaguchi, Yoshito Tsushima

**Affiliations:** 1 Department of Diagnostic Radiology and Nuclear Medicine, Gunma University Graduate School of Medicine, 39-22 Showa-machi 3-chome, Maebashi, 371-8511 Japan; 2 Department of Bioimaging Information Analysis, Gunma University Graduate School of Medicine, 39-22 Showa-machi 3-chome, Maebashi, 371-8511 Japan

**Keywords:** B-cell lymphoma, Anti-CD20 antibody, Radioimmunotherapy, Photoimmunotherapy

## Abstract

**Objective:**

Radioimmunotherapy (RIT) has proven effective for patients with relapsed and refractory lymphoma. However, new types of therapy are strongly desired as B-cell lymphoma remains incurable for many patients. Photoimmunotherapy (PIT) is an emerging targeted cancer therapy that uses photosensitizer (IR700)-conjugated monoclonal antibodies (mAbs) to specifically kill cancer cells. To evaluate the usefulness and potential role of PIT for treating B-cell lymphoma in a comparison with RIT, we performed in vivo PIT and RIT studies with an IR700 or ^90^Y-conjugated anti-CD20 mAb, NuB2.

**Methods:**

IR700 or ^90^Y were conjugated to NuB2. Since cell aggressiveness greatly affects the therapeutic effect, we selected both an indolent (RPMI 1788) and an aggressive (Ramos) type of B-cell lymphoma cell line. The in vitro therapeutic effect of PIT and the biodistribution profiles of IR700–NuB2 were evaluated. In vivo PIT and RIT studies were performed with 100 or 500 μg of IR700–NuB2 and 150 μCi/20 μg of ^90^Y-NuB2, respectively, in two types of B-cell lymphoma-bearing mice.

**Results:**

The in vitro studies revealed that Ramos was more sensitive than RPMI 1788 to PIT. The therapeutic effect of PIT with 500 µg IR700–NuB2 was superior to any other therapies against aggressive Ramos tumors, whereas RIT showed the highest therapeutic effect in indolent RPMI 1788 tumors. Since the uptake levels and intratumoral distribution of IR700–NuB2 were comparable in both tumors, a possible cause of this difference is the tumor growth rate. The PIT with 500 µg (IR700–NuB2) group showed a significantly greater therapeutic effect than the PIT with 100 µg group due to the higher and more homogeneous tumor distribution of IR700–NuB2.

**Conclusions:**

PIT was effective for both indolent and aggressive B-cell lymphoma, and the higher dose provided a better therapeutic effect. In aggressive tumors, PIT was more effective than RIT. Thus, PIT would be a promising strategy for the locoregional treatment or control of B-cell lymphoma. Since PIT and RIT have distinctive advantages over each other, they could play complementary rather than competitive roles in B-cell lymphoma treatment.

## Introduction

Lymphoma is the most common hematological malignancy with more than 450,000 new cases per year and 225,000 cancer deaths per year worldwide [1]. Lymphoma is a diverse group of B-cell tumors, T-cell tumors, and natural-killer cell tumors, and the majority of lymphomas (approx. 90%) are of B-cell origin [2]. The cornerstone of B-cell lymphoma treatment is chemotherapy as a single agent or in combination with immunotherapy and radiation therapy, which provide a great effect in many cases. However, the relapse rate of B-cell lymphoma is usually high, and sometimes recurring tumors obtain resistance to these conventional treatments. Thus, novel targeted therapies have been developed in preclinical and clinical practice.

Radioimmunotherapy (RIT) with tumor-specific monoclonal antibodies (mAbs) has proven effective for patients with relapsed and refractory lymphoma [3]. In RIT, mAbs are coupled to radioisotopes for delivering cytotoxic radiation exposure specifically to lymphoma cells. Two commercially available radiolabeled anti-CD-20 mAbs, ^90^Y-ibritumomab tiuxetan (Zevalin^®^) and ^131^I-tositumomab (Bexxar^®^), demonstrated superior therapeutic response compared to mAb alone [4]. Although RIT showed a potential for the treatment of aggressive lymphoma [5], it is currently approved only for ‘indolent’ (i.e., slow-growing) lymphoma. Tumor recurrence is observed in approximately half of responders within 1 year after treatment even among indolent lymphomas, although the response rate to RIT is as high as 70% [6]. Thus, B-cell lymphoma remains incurable for many patients, and new and more effective types of therapy are strongly desired.

Photoimmunotherapy (PIT) is an emerging targeted cancer therapy that uses mAbs as a vehicle to deliver a cytotoxic agent specifically to the tumor, as in RIT [7]. PIT uses mAbs conjugated with a photosensitizing near-infrared (NIR) phthalocyanine dye, IRDye700DX (IR700). By irradiating NIR light to a tumor, the photosensitizer-conjugated antibody-bound target cells are specifically killed. PIT is relatively safe compared to external radiotherapy and RIT because NIR light itself is harmless and the photosensitizer-conjugated antibody causes no toxicity without activation by NIR light. In animal studies, PIT showed a very promising therapeutic effect on many types of tumors including Burkitt’s lymphoma [8–12]. An early-phase clinical trial of PIT is being conducted in patients with head and neck cancers. However, further research is needed to assess the usefulness of PIT for B-cell lymphoma, since PIT for indolent lymphoma has not been evaluated and no comparative study with existing methods has been performed.

Both RIT and PIT use anti-tumor mAbs for delivering a cytotoxic agent (a radionuclide or photosensitizer) specifically to the tumor, but no study has compared and contrasted the effectiveness of RIT and PIT. In this study, we performed in vivo RIT and PIT experiments with ^90^Y- or IR700-conjugated anti-CD20 mAbs. Since cell aggressiveness plays a role in therapeutic effects, we selected both an indolent-type and an aggressive-type B-cell lymphoma cell line for tumor xenografts. Based on our results, we discuss the usefulness and potential role of PIT for B-cell lymphoma in comparison with RIT.

## Materials and methods

### Reagents

IRDye700DX (IR700) NHS ester was obtained from LI-COR Biosciences (Lincoln, NE, USA). A murine anti-CD20 monoclonal antibody, NuB2, was kindly supplied by Immuno-Biological Laboratories Co. (Takasaki, Japan). All other chemicals were of reagent grade.

### Synthesis of IR700–NuB2

The conjugation of IR700 NHS ester to NuB2 was performed as described [7]. In brief, NuB2 (1 mg/500 μl, 6.7 nmol) was incubated with IR700 NHS ester (65 μg, 33.3 nmol) in 0.1 M Na_2_HPO_4_ (pH 8.5) at room temperature for 2 h. The reaction mixture was purified with a Sephadex G25 column (PD-10; GE Healthcare, Piscataway, NJ, USA). The mAb concentration was determined with a NanoDrop 1000 Spectrophotometer (Thermo Scientific, Wilmington, DE, USA) by measuring the absorption at 280 nm. The concentration of IR700 was measured by absorption at 689 nm to confirm the number of fluorophore molecules per mAb. The synthesis was controlled to attach approximately 3–4 IR700 molecules to a single antibody.

### Synthesis of radiolabeled NuB2

2-(4-Isothiocyanatobenzyl)-diethylenetriaminepentaacetic acid (SCN-Bn-DTPA; Macrocyclics, Dallas, TX, USA) was used for labeling NuB2 with ^90^Y. SCN-Bn-DTPA in dimethylformamide was added to NuB2 at 5 mg/ml in 50 mM borate-buffered saline (pH 8.5) at the molar ratio of 5:1. After incubation at 37 °C for 24 h, DTPA–NuB2 was purified using a Bio-Spin column (Bio-Rad Laboratories, Hercules, CA, USA). For radiolabeling, 25–50 μl of a solution of ^90^YCl_3_ (37 MBq, Nuclitec, Braunschweig, Germany) was incubated with 50–100 μl of 0.25 M acetate buffer (pH 5.5) for 5 min at room temperature, followed by incubation with 100 μg of DTPA–NuB2 for 1 h at 40 °C. The ^90^Y-labeled antibody was purified using a Bio-Spin column or PD-10 column. The radiochemical purity of ^90^Y-NuB2 was confirmed as >95% by Tec-Control Chromatography Strips (Biodex Medical Systems, Shirley, NY, USA) developed with saline. Iodine-125-labeled antibodies were prepared according to standard protocols for the chloramine-T method.

Briefly, 740 kBq/2 μl of Na^125^I (PerkinElmer, Waltham, MA, USA) and 1 μg of chloramine-T in 1 μl of 0.3 M phosphate buffer were added to 40 μg of mAb in 100 μl of 0.3 M phosphate buffer. The ^125^I-labeled mAb was purified using a Bio-Spin column.

### Cell culture

We obtained the aggressive B-cell lymphoma cell line Ramos (Burkitt’s lymphoma) and the indolent B-cell lymphoma cell line RPMI 1788 (B lymphoblast) from the American Type Culture Collection (ATCC, Manassas, VA, USA). Cells were grown in RPMI 1640 medium (Wako Pure Chemical Industries, Osaka, Japan) supplemented with 10% heat-inactivated fetal bovine serum (Japan Bioserum, Hiroshima, Japan) and 1% penicillin/streptomycin (Wako Pure Chemical Industries) in tissue culture flasks.

### Cell binding assay

A solution of ^125^I-NuB2 (containing 0.01 µg of antibody) was added to 100 µl of cell suspension of Ramos or RPMI 1788 (2 × 10^6^) cells with various amounts of nonlabeled antibody. After incubation for 1 h at room temperature, the cell suspension was centrifuged at 3000 rpm for 5 min. After the supernatant was removed, the radioactivity of the cell fraction was measured with a well-type γ-counter (ARC7001; Hitachi Aloka Medical, Tokyo, Japan) and compared with the initial radioactivity. *B*_max_ (antigen expression level) and *K*_d_ values were determined by a Scatchard plot analysis. The immunoreactivity of DTPA–NuB2 or IR700–NuB2 was evaluated by the same methods using ^125^I-DTPA–NuB2 and ^125^I-IR700–NuB2, respectively.

### In vitro PIT

One hundred thousand cells were seeded into 12-well plates with phenol red-free RPMI 1640 medium (Wako Pure Chemical Industries) and incubated overnight. IR700–NuB2 was then added to the culture at the final concentration of 10 µg/ml. The cultures were incubated for 1 h. After being washed with phosphate-buffered saline (PBS), the cells were suspended again in the phenol red-free medium. We divided the cell suspensions into one control group and four treatment groups. The treatment groups were irradiated with an NIR light-emitting diode (Marubeni America, Santa Clara, CA, USA), which emits light at the range 680–700 nm wavelength with different energy density values: 0 (IR700–NuB2 only), 2, 5, and 10 J/cm^2^ (irradiation time was 40, 100 and 200 s, respectively). The energy density was measured by optical power meter (PM 100; Thorlabs, Newton, NJ, USA). For the determination of the cytotoxic effects of PIT, at 1 h after the light exposure, propidium iodide (PI; Dojindo, Kumamoto, Japan) was added to the cell suspension at the final concentration of 2 µg/ml and incubated at room temperature for 15 min. Flow cytometry was performed with an Attune Acoustic Focusing Cytometer (Applied Biosystems, Foster City, CA, USA). The percentage of dead cells in the treatment group was obtained by subtracting the mean percentage of dead cells in the corresponding control group from the measured value.

### Animal and tumor models

The animal experiments were conducted in accordance with our institutional guidelines and were approved by the institutional Animal Care Committee. Six- to eight-week-old female KSN nude mice (Japan SLC, Shizuoka, Japan) were inoculated with 3 × 10^6^ of Ramos cells or 1 × 10^7^ of RPMI 1788 cells. Mice were monitored every 2 days for their general health and tumor volumes.

For the determination of the tumor volume, the length and the width were measured with an external caliper. Tumor volumes were determined by the following formula: tumor volume = length × width^2^ × 0.5. In vivo studies were performed after the tumor volume reached approximately >200 mm^3^. The average tumor volume of the Ramos and RPMI 1788 tumors for the therapeutic studies were 335.8 ± 3.8 and 312.3 ± 3.7 mm^3^, respectively.

### In vivo PIT and RIT

Mice were randomly assigned to five groups of at least five animals per group. These five groups were: (1) the control group (no treatment), (2) the Ab-only group (IR700–NuB2 100-μg injection without NIR light exposure), (3) the PIT100 group (PIT with IR700–NuB2 100-μg injection), (4) the PIT500 group (PIT with IR700–NuB2 500-μg injection), and (5) the RIT group (^90^Y-NuB2 150 μCi/20-μg injection). NIR light was administered on day 1 and day 2 after the injection of IR700–NuB2 with the energy density values 50 J/cm^2^ (irradiation time 17 min) and 100 J/cm^2^ (irradiation time 34 min), respectively. Mice were anesthetized with isoflurane during the procedure. Serial fluorescence images, as well as white light images, were obtained before and after each NIR light exposure (day 1 and day 2) using a Maestro In vivo Imaging System (CRi, Woburn, MA) with an excitation filter at 671–705 nm and the emission filter at 700 nm longpass. The images were analyzed using ImageJ ver. 1.50i software [13]. The mice were then monitored every 2 days for their general health and tumor volumes. After 30 days or when a tumor volume reached 2000 mm^3^, the mice were euthanized. The relative tumor size was calculated by dividing the tumor volume by each initial tumor volume. A complete response (CR) was assigned when the relative tumor size became <0.1 and had not increased within 30 days. A partial response (PR) was assigned when the relative tumor size became <0.7. The time to tumor progression was defined as the time when the relative tumor size reached 2.5 [14].

### Biodistribution studies

Biodistribution studies were performed when the tumor size became similar to that in the therapeutic studies described above. A mixture of ^125^I-IR700–NuB2 and cold IR700–NuB2 (10 kBq, protein dose: 100 or 500 μg) was injected via the tail vein into tumor-bearing mice, and the biodistribution studies were performed at 24 h after the injection. Organs of interest were excised and weighed, and the radioactivity was measured with a well-type γ-counter. The uptake data were calculated as the percentage-injected dose per gram of tissue (%ID/g).

### Fluorescence microscopy studies

Tumor xenografts were excised from nude mice 24 h after the injection of IR700–NuB2 (100 or 500 μg) and embedded in the OCT compound. Frozen sections (80 μm thick) were prepared and fluorescence was assessed by fluorescence microscopy (BZ9000; Keyence, Osaka, Japan). Hematoxylin and eosin (HE) staining was then performed.

### Statistical analysis

Data are expressed as the mean ± standard error of the mean (SEM). Statistical analyses were carried out using IBM SPSS Statistics 22 software (IBM, Armonk, NY, USA) and R Programming Language ver. 3.3.2 software. We used unpaired *t* tests to compare differences between pairs of groups. Differences in the relative tumor size at day 8 and day 14 among the groups were evaluated by one-way ANOVA followed by Tukey’s multiple comparison to compare the effectiveness of the different treatments. We used Spearman’s rank correlation coefficient to analyze the correlation between the dead cell ratio and the power intensity of NIR light. The Wilcoxon signed rank test was used to test the differences in fluorescence intensity between the PIT100 and PIT500 groups. Values of *p* < 0.05 were considered significant.

## Results

### Binding assay

The binding of ^125^I-NuB2 to Ramos and RPMI 1788 cells was inhibited by NuB2 in a concentration-dependent manner. The calculated CD20 expression levels of the Ramos and RPMI 1788 were approximately 1.02 × 10^5^ and 1.28 × 10^5^ molecules per cell, respectively. ^125^I-DTPA–NuB2 and ^125^I-IR700–NuB2 also showed binding to cells and was inhibited by each of the NuB2 conjugates. The calculated Kd values of NuB2, DTPA–NuB2 and IR700–NuB2 toward Ramos cells were approximately 2.69 × 10^−9^, 5.54 × 10^−9^, and 5.33 × 10^−9^ M, respectively, indicating that DTPA or IR700 conjugation has little effect on the immunoreactivity of NuB2.

### In vitro PIT

**Fig. 1:**
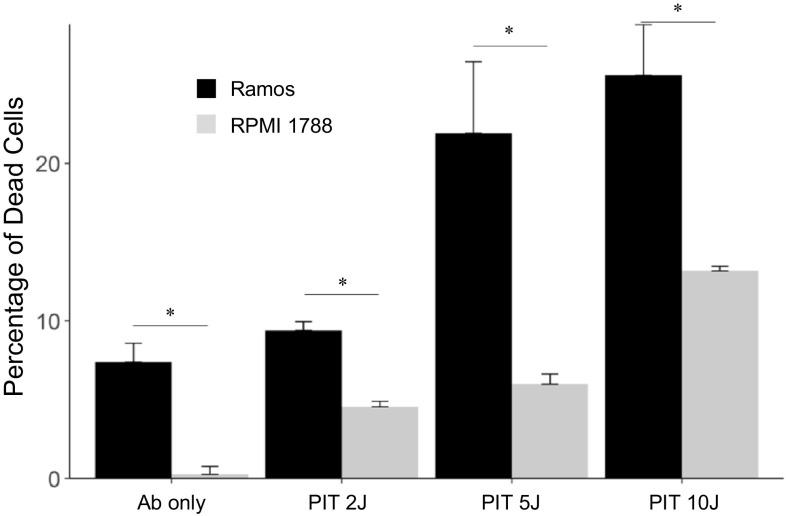
The percentage of dead cells after treatment with PIT in vitro. The data are mean ± SEM. There are significant differences between dead cells in Ramos and RPMI 1788 cells at the same light intensity (*p < 0.05)

The percentage of cell death was significantly correlated with the NIR light dose for both the Ramos (*r* = 0.88, *p* < 0.05) and RPMI 1788 cells (*r* = 0.90, *p* < 0.05). We found no significant difference between the control group and the group treated with IR700–NuB2 only. The percentage of cell death of Ramos cells was significantly higher than that of RPMI 1788 cells at the same light intensity (Fig. 1). For example, when exposed to 10 J of NIR light, the percentage of dead Ramos cells was approximately 26 ± 3.2%, whereas that in the RPMI 1788 cells was only 13 ± 0.3%.

### In vivo PIT and RIT in Ramos tumor-bearing mice

**Fig. 2:**
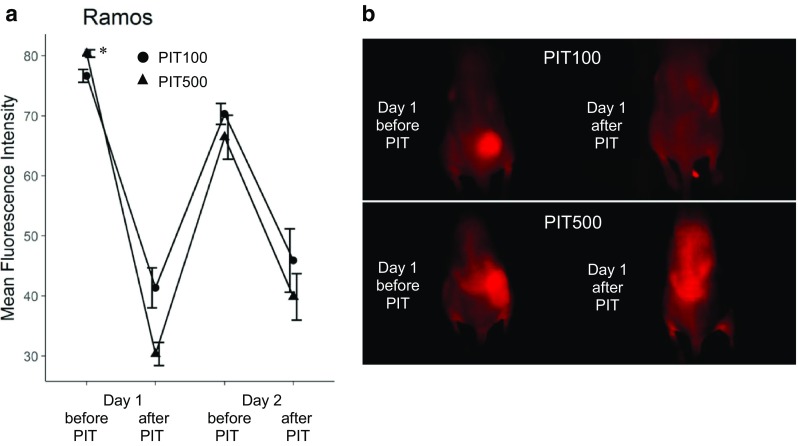
In vivo fluorescence imaging of Ramos tumor-bearing mice. **a** Tumor fluorescence intensity before and after treatment with PIT in the PIT100 and PIT500 groups. The data are mean ± SEM. The fluorescence intensity of the PIT500 group was significantly higher than that of the PIT100 group on day 1 before PIT (*p = 0.012). **b** Typical fluorescence image on day 1 before and after PIT in the PIT100 group and PIT500 group

One day after the injection of IR700–NuB2, the tumor showed high fluorescence intensity (Fig. 2). After the first exposure to NIR light, the fluorescence signal was decreased due to the washout of IR700–NuB2 from dead cells and partial photobleaching (Fig. 2a). IR700–NuB2 re-accumulated in the tumors by the time point before the second NIR light exposure, though the intensity was lower than that before the first NIR light exposure. The fluorescence intensity of the PIT500 group was significantly higher than that of the PIT100 group (*p* = 0.012), suggesting that as the dose of IR700–NuB2 increased, the tumor accumulation of it increased.

**Fig. 3:**
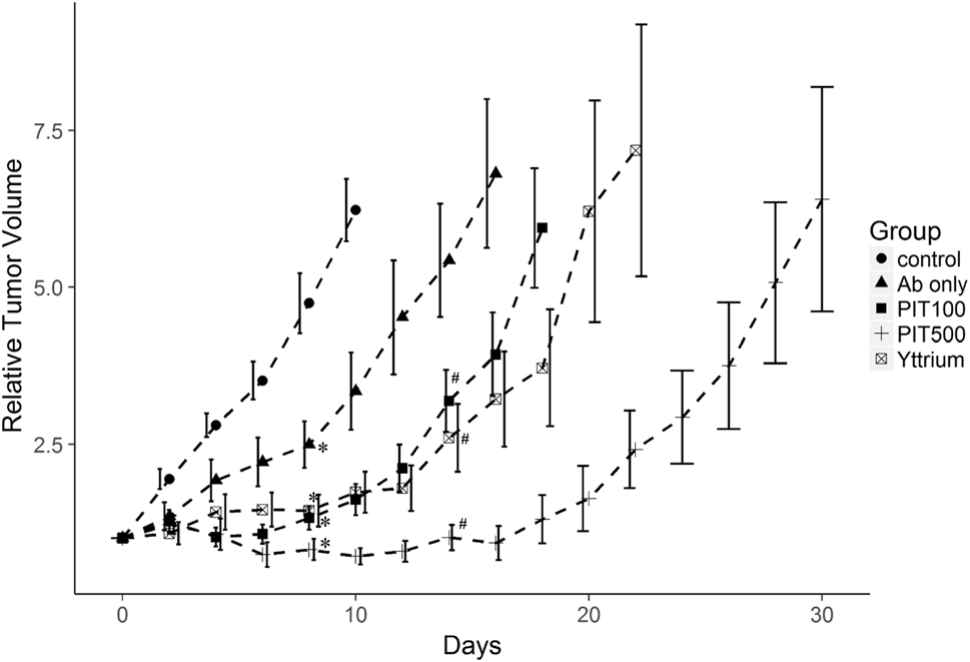
Relative tumor volume after the treatment of Ramos tumor-bearing mice. The data are mean ± SEM. Tumor growth was significantly inhibited in all treated groups compared to the untreated control group on day 8 (*p < 0.05). Tumor inhibition was significantly improved in the mice treated with PIT or RIT compared to mice treated with Ab only on day 14 (^#^ *p* < 0.05)

Tumor growth was significantly inhibited in all four treatment groups compared to the control group (Fig. 3). Treatment with PIT (PIT100 and PIT500) and RIT resulted in significantly better results compared to IR700–NuB2 alone (Ab only) on day 14 (*p* < 0.05). The time to tumor progression of the PIT500 group was significantly longer than that of the RIT or PIT100 groups (25 ± 0.81, 16 ± 0.80 and 14 ± 0.40 days, respectively, *p* < 0.05). Importantly, five of the six mice in the PIT500 group and three of the eight mice in the PIT100 group showed PRs after treatment, whereas no mice in the RIT or Ab-only group showed tumor size reduction.

### In vivo PIT and RIT in RPMI 1788 tumor-bearing mice

**Fig. 4:**
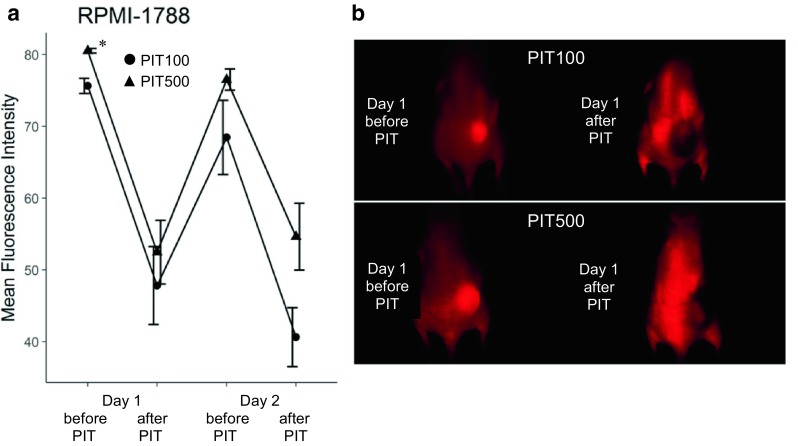
In vivo fluorescence imaging of RPMI 1788 tumor-bearing mice. **a** Tumor fluorescence intensity before and after treatment with PIT in the PIT100 group and PIT500 group. The data are mean ± SEM. The PIT500 group’s fluorescence intensity was significantly higher than that of the PIT100 group on day 1 before PIT (*p = 0.001). **b** Typical fluorescence image on day 1 before and after PIT in the PIT100 and PIT500 groups

Similarly to the Ramos tumors, IR700–NuB2 accumulated in the RPMI 1788 tumors at 1 day after the injection, and the fluorescence signal decreased by PIT treatments (Fig. 4). The accumulation of IR700–NuB2 in the PIT500 group was significantly higher than that in the PIT100 group (*p* = 0.001).

**Fig. 5:**
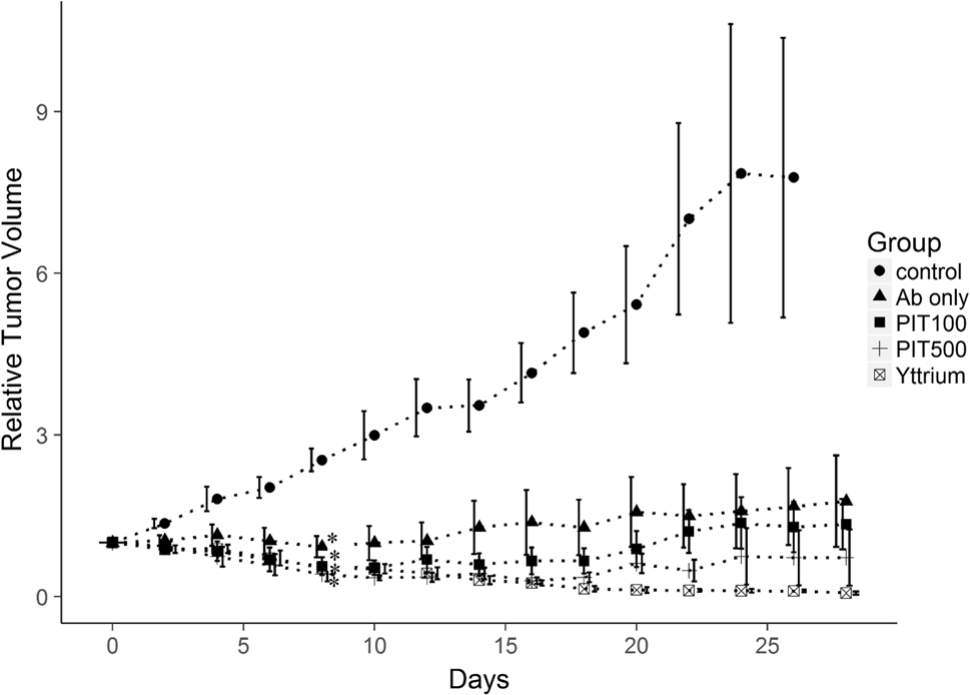
Relative tumor volume after the treatment of RPMI 1788 tumor-bearing mice. The data are mean ± SEM. Tumor growth was significantly inhibited in all treated groups compared to the untreated control group on day 8 (*p < 0.05)

On average, the time to tumor progression of the RPMI 1788 control group was much slower than that of the Ramos control group (10.7 ± 0.66 and 4.5 ± 0.22 days, respectively, *p* < 0.01). Tumor growth was significantly inhibited in all four treatment groups compared to the control groups (Fig. 5). The response rates to each treatment are summarized in Table 1. RIT treatment was the most effective compared to the other groups, and all mice in the RIT groups achieved a CR after treatment. The effectiveness of treatment in the PIT500 group was better than that of the PIT100 group. The effectiveness of PIT and RIT was more prominent in the RPMI 1788 tumors compared to the Ramos tumors.

**Table 1:** Response rates of RPMI 1788 tumors to each treatment

	Response rate
Control (*n* = 6)	0%
Ab only (*n* = 6)	33.3% (CR: 2, PR: 0)
PIT100 (*n* = 6)	83.3% (CR: 2, PR: 3)
PIT500 (*n* = 6)	100% (CR: 4, PR: 2)
RIT (*n* = 6)	100% (CR: 6, PR: 0)

*CR* complete response, *PR* partial response

### Biodistribution and fluorescence microscopy results

**Fig. 6:**
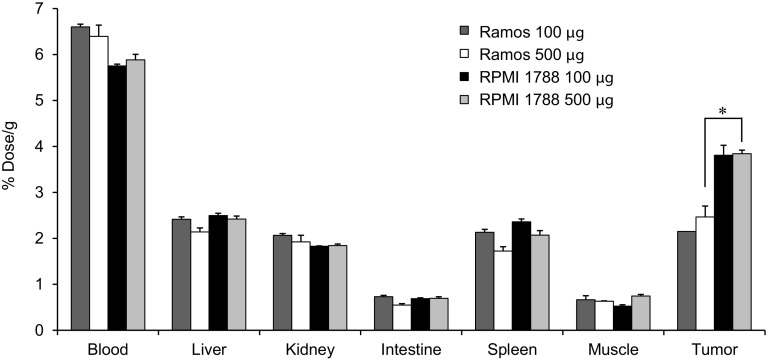
Biodistribution of ^125^I-IR700–NuB2 with 100 or 500 μg of cold IR700–NuB2 in Ramos or RPMI 1788 tumor-bearing mice 24 h after injection. Data were calculated as the percentage of injected dose per gram of tissue and are represented as the mean ± SEM (*n* = 4). The tumor accumulation level in the RPMI 1788 tumors was significantly higher than that in the Ramos tumors treated with an injection of 500 μg of cold IR700–NuB2 (*p < 0.05)

At 24 h after the injection, ^125^I-IR700–NuB2 showed accumulation in both the Ramos and RPMI 1788 tumors (Fig. 6). In both Ramos tumor-bearing mice and RPMI 1788 tumor-bearing mice, the tumor accumulation level of ^125^I-IR700–NuB2 (% dose/g) was not significantly different between the use of 100 and 500 μg of cold IR700–NuB2. These results indicated that the amount of IR700–NuB2 accumulated in the tumor was larger in the 500 μg-injected group than in the 100 μg-injected group, since the total protein amount could be estimated by multiplying the protein dose by the % dose. The accumulation level of ^125^I-IR700–NuB2 in the RPMI 1788 tumors was significantly higher than that in the Ramos tumors with the 500-μg dose of cold IR700–NuB2 injection (*p* < 0.05), but not significantly higher with the 100-μg dose.

**Fig. 7:**
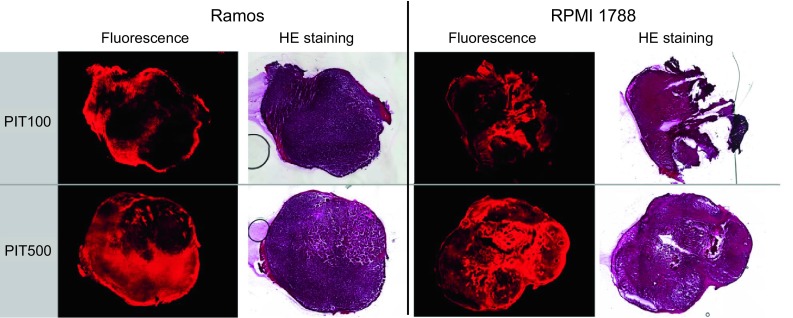
Fluorescence microscopy images of Ramos and RPMI 1788 tumors 24 h after an injection of IR700–NuB2. HE staining of the same section of the fluorescence image was also performed. *Upper row* injected with 100 μg of IR700–NuB2. *Lower row* injected with 500 μg of IR700–NuB2

Ex vivo fluorescence imaging demonstrated high accumulations of IR700–NuB2 in the tumors at 24 h after injection (Fig. 7). The intratumoral distributions of IR700–NuB2 in the 500 μg-injected group were more homogeneous compared to the 100 μg-injected group, throughout the tumors.

## Discussion

In this study, we selected the anti-CD20 antibody NuB2 and prepared an antibody–radionuclide or antibody–photosensitizer conjugate. Our experiments revealed that (1) PIT is effective for both types of B-cell lymphoma and (2) the therapeutic effect of PIT was better than that of RIT in aggressive lymphoma. In addition, PIT has a potential to treat larger tumors since a high-dose injection improves PIT’s therapeutic effect without inducing significant toxicity to normal organs. We observed herein that the therapeutic effect of PIT was better than that of RIT in aggressive tumors but not in indolent tumors.

In our in vitro and in vivo PIT studies, PIT showed a distinctive effect on Ramos and RPMI 1788 cell lines and mouse xenograft models. In the aggressive Ramos tumors, the therapeutic effect of PIT with 500-µg IR700–NuB2 was superior to any other therapeutic interventions including RIT, whereas in the indolent RPMI 1788 tumors, RIT showed the highest therapeutic effect. The effectiveness of PIT and RIT was more prominent in the RPMI 1788 tumors compared to the Ramos tumors. Although ^125^I-IR700–NuB2 showed significantly higher accumulations in the RPMI 1788 tumors compared to the Ramos tumors with 500 µg IR700–NuB2, the amount of IR700–NuB2 accumulated in the Ramos tumor with 500-μg injection would be larger than in the RPMI 1788 tumor with 100-μg injection. Nevertheless, the PIT100 group of RPMI 1788 tumors showed better therapeutic effects than the PIT500 group of Ramos tumors. In addition, IR700–NuB2 showed comparable intratumoral distributions in both tumor types. Thus, a possible cause of this difference of therapeutic effect is the differing properties of the two cell lines used, such as tumor growth rate and sensitivity, rather than the tumor accumulation levels of antibody.

None of the treatments was able to eliminate the Ramos tumors, in part because the tumors’ growth rate exceeded the cell death rate. Since both Ramos cells and RPMI 1788 cells have comparable expression levels of CD20 and since IR700–NuB2 has a high affinity to CD20, a similar amount of IR700–NuB2 would bind to both cell lines. However, PIT had a greater therapeutic effect in vitro on Ramos cells than RPMI 1788 cells. Thus, Ramos cells are more sensitive to PIT than RPMI 1788 cells. Therefore, although Ramos cells are more sensitive to PIT, the Ramos tumors started to regrow earlier compared to the RPMI 1788 tumors, which is likely to be simply because of the Ramos tumors’ faster growth rate.

To induce phototoxicity by PIT, a certain amount of IR700–mAb should bind to the target [7]. The cytotoxic effect of PIT can be enhanced by increasing the level of bound IR700–mAb [15]. However, due to the heterogeneity in the expression levels of CD20 and the physical inaccessibility to IR700–NuB2, it would be difficult to deliver an adequate amount of IR700–NuB2 to every cell. Thus, because of the heterogeneous distribution of IR700–NuB2, a minimal number of cells that escaped immediate cell death after NIR light exposure would have survived and started to regrow. Once an adequate number of IR700–NuB2 is delivered to all cells, PIT would be the more effective therapy not only for indolent but also for aggressive B-cell lymphoma.

We employed a 5× higher dose of IR700–NuB2 (500 µg) in addition to the conventional dose (100 µg) because we used tumors that were >4× larger (>200 mm^2^) compared to the tumors described in previous reports (<50 mm^2^) [7, 12, 16]. Consistent with an earlier study [7], in our experiment the frozen sections in the PIT500 group revealed higher and more homogeneous distributions of IR700–NuB2 in the tumors compared to the PIT100 group. As a result, the PIT500 group showed a significantly greater therapeutic effect compared to the PIT100 group. Unlike the use of a radiolabeled mAb where normal organ toxicity restricts the higher-dose injection, high-dose injection is possible for PIT because IR700–mAb itself causes no phototoxicity without NIR light exposure. Our results thus indicate that PIT with the higher dose has the potential to treat tumors larger than those previously reported. Even if IR700–mAb is unable to reach the deeper part of a tumor at first, repeated light exposure would be beneficial to treat the whole tumor. Since PIT can increase the permeability of tumor vessels (as the superenhanced permeability and retention (SUPR) effect [17, 18]), a high amount of IR700–mAb remaining in the circulation will be delivered into the deep part of the tumor after NIR light exposure.

The tumor distribution of ^90^Y-NuB2 would be heterogeneous since the protein dose was low (20 µg). However, since RIT has a crossfire effect, ^90^Y-NuB2 showed a therapeutic effect in both lymphoma models. Based on specific activity of ^90^Y-NuB2, protein dose of 150 µCi ^90^Y-NuB2 was adjusted to 20 µg. Although the distribution pattern of the antibody in the tumor would be improved by increasing the protein dose, the excess amount of unlabeled protein would decrease the tumor accumulation level of the radiolabeled protein [19], which leads to the decrease in the therapeutic effect. Higher dose of radioactivity would improve the therapeutic effect of RIT; however, it is known that the maximum tolerated dose for tumor-bearing mice is less than 200 µCi. Indeed, in our previous RIT studies, some mice have died after 200 µCi injection due to the radiotoxicity [20]. Therefore, we decided to use 150 µCi in this study. Consistent with the accumulated clinical evidence, RIT greatly suppressed the tumor growth rate of indolent RPMI 1788 tumors compared with the aggressive Ramos tumors. Since the therapeutic effect of RIT depends on the total radiation dose, the Ramos cells could grow before a lethal dose of radiation was delivered, which led to the relatively early regrowth of the Ramos cells. On the other hand, since the growth rate of the RPMI 1788 cells was slow, there was enough time to deliver a lethal dose of radiation to all cells. For indolent lymphomas, RIT should be selected prior to PIT, because of RIT’s excellent therapeutic effect and convenience (it requires only a single injection). However, PIT might be an option for large tumors for which the outcome of RIT is inadequate [21].

The limited tissue penetration of NIR light (approximately 2 mm) is the main disadvantage of PIT. In many lymphoma cases, PIT may not be a feasible approach because these tumors are usually located in deep tissues. However, a light fiber can be extended to a lymphoma located in deep tissues with the use of a device such as an endoscope, laparoscope, or image-guided percutaneous needle. Although limited penetration may also be a significant challenge for large tumors, a light fiber could potentially treat a lesion as large as a cylinder-shaped area with a diameter of 4 cm and a height of the fiber’s length. Thus, with the use of multiple light fibers, we can expose the entire region of large tumors. Since PIT requires NIR light exposure to each lesion, it might be perceived as a local therapy rather than systemic therapy. However, PIT causes cell rupture and release of many molecules from the cytosol and nucleus which could induce maturation of the dendritic cells and activating a patient’s innate immune system [22], and it thus may be able to kill entire cancer cells distributed throughout the body. Another possible treatment for disseminating tumors is combination therapy with other systemic therapy like chemoradiation therapy, because PIT can cause a rapid and strong therapeutic effect against locoregional tumors without inducing significant toxicity to normal organs.

To our knowledge, this is the first report that directly compared the therapeutic effects of PIT and RIT. Our findings indicate that PIT and RIT are complementary rather than competitive. PIT, RIT or both should be selected on a case-by-case basis for patients considering the characteristics of each tumor such as its type, size, number, site, and more. Although the precise differences in the therapeutic effects of PIT and RIT for other tumor types remain to be established, the present study demonstrated that PIT and RIT can be complementary treatments.

## Conclusion

Photoimmunotherapy was effective for both indolent and aggressive B-cell lymphomas, and PIT with a high dose of IR700–NuB2 showed a better therapeutic effect and potential to treat large tumors. The therapeutic effect of PIT was better than that of RIT against aggressive tumors, but not indolent tumors. PIT could thus be a promising strategy for the locoregional treatment of lymphomas. Since PIT and RIT each has unique advantages, they could play complementary rather than competitive roles in B-cell lymphoma treatment.
